# Machine-Learning-Based Genomic Prioritization and Genome-Wide Association Analysis of Growth Traits in Central Anatolian Merino Sheep

**DOI:** 10.3390/ani16203243

**Published:** 2026-10-15

**Authors:** Yunus Arzik, Mehmet Kizilaslan, Sedat Behrem, Mehmet Ulaş Çınar

**Affiliations:** 1 Department of Animal Science, Faculty of Veterinary Medicine, Aksaray University, 68100 Aksaray, Türkiye; yunus.arzik@aksaray.edu.tr (Y.A.); sedatbehrem@aksaray.edu.tr (S.B.); 2 Department of Animal and Dairy Sciences, University of Wisconsin-Madison, Madison, WI 53706, USA; kizilaslan@wisc.edu; 3 Department of Animal Science, Faculty of Agriculture, Erciyes University, 38280 Kayseri, Türkiye; 4 Department of Veterinary Microbiology & Pathology, College of Veterinary Medicine, Washington State University, Pullman, WA 99164, USA; 5 Betül-Ziya Eren Genome and Stem Cell Research Center (GENKÖK), Erciyes University, 38280 Kayseri, Türkiye

**Keywords:** *Ovis aries*, growth traits, machine learning, genomic prediction, genome-wide association study, SNP prioritization

## Abstract

Growth traits are major economic drivers in sheep production, yet their genetic architecture is highly polygenic and influenced by multiple genetic and environmental factors. Here, we integrated machine-learning-based genomic prioritization and prediction with conventional mixed-model genome-wide association analysis to investigate growth traits in Central Anatolian Merino sheep. Phenotypic records for birth weight (BW), weaning weight (WW), six-month weight (W6), and average daily gains before (ADWG1) and after weaning (ADWG2) were obtained from 278 lambs genotyped using the Illumina OvineSNP50 BeadChip. Following genotype quality control, 275 animals and 46,902 SNPs were retained for genome-wide analyses. Machine-learning models were evaluated using five-fold outer cross-validation repeated five times. Within each outer training set, SNP preprocessing, feature prioritization, consensus ranking, marker-panel construction, hyperparameter optimization, and model fitting were performed independently. LASSO, Elastic Net, Random Forest, and XGBoost were used for SNP prioritization, and 50-, 100-, and 200-SNP panels were evaluated using Ridge regression, Random Forest, XGBoost, and Support Vector Regression. Conventional mixed-model GWAS identified no SNP reaching genome-wide significance after Bonferroni or FDR correction, although several suggestive and chromosome-wide signals were detected. Exploratory comparison of ML prioritization and GWAS results revealed concordant genomic signals for related traits, particularly ADWG1–WW and ADWG2–W6; these were interpreted as shared genomic signals rather than evidence of pleiotropy. Candidate gene annotation identified biologically relevant positional candidate genes involved in muscle development, skeletal growth, metabolism, and cellular regulation, including *PRMT1*, *TNC*, *POU1F1*, *RASD2*, *ERCC6L2*, *SSH2*, and *DYM*. Overall, the complementary use of machine-learning-based genomic prioritization and conventional GWAS provided a conservative framework for investigating the genomic architecture of growth traits. The identified genomic signals and positional candidate genes should be considered exploratory and require validation in larger and independent sheep populations before their potential application in genomic selection.

## Simple Summary

This study investigated the genetic basis of growth traits in Central Anatolian Merino sheep using machine-learning-based genomic prioritization and prediction together with conventional genome-wide association analysis. A total of 278 lambs were evaluated for birth weight, weaning weight, six-month weight, and average daily gain before and after weaning. Machine-learning models were evaluated using repeated nested cross-validation, in which SNP prioritization, marker-panel construction, model tuning, and prediction were performed within the training data. LASSO, Elastic Net, Random Forest, and XGBoost were used for SNP prioritization, while Ridge regression, Random Forest, XGBoost, and Support Vector Regression were evaluated for genomic prediction using 50-, 100-, and 200-SNP panels. Predictive performance was strongest for birth weight and pre-weaning average daily gain and was lower for later growth traits. Conventional mixed-model GWAS was additionally performed to provide SNP-wise association evidence; although no SNP reached genome-wide significance after multiple-testing correction, several suggestive and chromosome-wide signals were observed. Shared SNPs were mainly found between closely related growth stages and were interpreted as shared genomic signals rather than evidence of pleiotropy. Important positional candidate genes included *PRMT1*, *TNC*, *POU1F1*, *RASD2*, *ERCC6L2*, *SSH2*, and *DYM*, which have reported biological functions related to muscle development, skeletal growth, metabolism, and cellular regulation. Overall, the complementary use of machine-learning-based genomic prioritization and conventional GWAS identified exploratory genomic signals and positional candidate genes that warrant validation in larger and independent sheep populations.

## 1. Introduction

Sheep (*Ovis aries*) are an essential element of the worldwide livestock industry, significantly enhancing food security, rural economies, and international commerce. Their significance stems from their multifunctional usage, adaptability to various agroecological zones, and economic worth [1]. The species’ resilience to extreme environmental conditions highlights its worldwide significance. Their resilience promotes the economic feasibility of sheep breeding while underscoring its sustainability and efficiency. Sheep can be cultivated in arid, semi-arid, and hilly locations that are inappropriate for intensive agriculture, hence supporting food production in marginal areas [1]. Lamb meat is rich in protein, vitamin B12, essential amino acids, and essential fatty acids [2,3]. The nutritional attributes of lamb meat may satisfy contemporary customers’ demand for healthful meat. Lamb meat has gained significant popularity globally during the past two decades [4]. The global production of lamb meat rose from 11.28 million tons to 16.03 million tons between 2000 and 2020 [4]. Body weight is the paramount index for growth and development in sheep production, directly and indirectly affecting meat and wool yield, as well as reproductive performance. In production, the weights at birth, weaning, yearling, and adulthood are utilized to indicate the growth and development of sheep. The increase in body weight is determined by a mix of the sheep’s genetic predisposition and its nutrition and management practices. Weight gain is a significantly heritable characteristic and serves as a primary criterion for selection [5,6]. The Central Anatolian Merino is a dual-purpose sheep population developed through crossbreeding German Mutton Merino with the indigenous Akkaraman and is mainly raised for meat and wool production under Central Anatolian conditions. Previous studies have reported considerable phenotypic and genetic variation in growth traits in this population, including birth weight, weaning weight, and average daily gain. These characteristics make growth performance an important breeding objective in Central Anatolian Merino sheep and provide a relevant basis for investigating the genomic architecture underlying growth at different developmental stages [5].

Growth traits are classified as quantitative traits affected by numerous genes, and quantitative trait loci (QTL) can clarify aspects of this trait variance. Recent years have witnessed significant progress in identifying candidate genes that affect sheep body weight through GWAS and other biological techniques [7,8,9]. A GWAS employs millions of single-nucleotide polymorphisms (SNPs) as molecular genetic markers to perform control or correlation analyses over the entire genome, therefore examining the genetic mutations associated with complex phenotypes [10]. This technique has been utilized to assess the SNPs associated with the principal attributes of farm animals. Traditional GWAS employs hypothesis-driven statistical models, generally assessing each single-nucleotide polymorphism (SNP) independently for its connection with a phenotype through linear or logistic regression frameworks [11] and mixed linear models are extensively utilized to account for population structure and relatedness [12]. These methodologies produce interpretable results, including *p*-values and Manhattan plots, which aid in locus identification. Nevertheless, they presume additive, independent SNP effects and may be insufficiently powerful to identify epistatic or nonlinear genetic interactions, particularly in polygenic characteristics [11]. Conversely, machine-learning approaches provide a data-driven framework for evaluating complex genotype–phenotype relationships and prioritizing informative genomic features [13]. Algorithms including random forests (RFs), support vector machines (SVMs), gradient boosting, and Elastic Net have been progressively utilized in genomic data analysis [14,15]. Unlike conventional GWAS, which provides SNP-wise statistical tests of association, machine-learning approaches primarily focus on prediction and feature prioritization. Therefore, measures of feature importance derived from machine-learning models should not be interpreted as equivalent to statistical significance obtained from conventional GWAS [16].

This study advances previous work by integrating machine-learning-based genomic prioritization and prediction with conventional genome-wide association analysis to investigate the genetic architecture of growth traits in Central Anatolian Merino sheep. Five traits representing different growth stages were evaluated: birth weight (BW), pre-weaning average daily gain (ADWG1), weaning weight (WW), post-weaning average daily gain (ADWG2), and six-month body weight (W6). Machine-learning models were evaluated using repeated nested cross-validation, with SNP prioritization, marker-panel construction, model tuning, and prediction performed within the training data, while mixed-model GWAS was conducted separately to provide SNP-wise association evidence. This complementary framework was used to prioritize genomic signals and positional candidate regions associated with growth performance in Central Anatolian Merino sheep.

## 2. Materials and Methods

Blood sample collection adhered to the ethical standards set by the Committee for the Purpose of Control and Supervision of Experiments on Animals (HADYEK), and the procedure was approved by the Institutional Animal Ethics Committee of Erciyes University (Approval No. 191, dated 21 November 2022).

### 2.1. Animals and Phenotypic Data

The experimental cohort consisted of 278 randomly selected Central Anatolian Merino (CAM) lambs, including 81 males and 197 females, obtained from three large-scale commercial farms located in the vicinity of Ankara province, Türkiye. The CAM is a composite Merino-type breed developed in Central Anatolia through crossbreeding of imported Merino lines (primarily German and Australian origin) with the indigenous fat-tailed Akkaraman breed [17]. The study was conducted under continental climatic conditions characterized by cold winters and hot, dry summers, resulting in limited pasture quality. All animals were enrolled in the National Community-Based Small Ruminant Breeding Program, where selective breeding has been applied across generations based on growth performance, with top-performing rams and ewes systematically mated. Lambing occurred between January and February, and animals were weaned between April and May at an average age of approximately three months.

Phenotypic records included birth weight (BW), weaning weight (WW), and body weight at 180 days (W6). Additionally, average daily gain before weaning (ADWG1) and post-weaning (ADWG2) were calculated. Weaning weight was standardized to 90 days, and six-month weight was adjusted to 180 days using linear interpolation. Blood samples (~6 mL) were collected via jugular venipuncture into EDTA-coated vacutainer tubes immediately after phenotypic recording. Samples were transported under cold chain conditions to the Genetics Laboratory of the International Center for Livestock Research and Training (Ankara, Türkiye), where DNA extraction and genotyping were performed. Raw phenotypic observations, rather than phenotypes pre-adjusted for environmental effects, were used in the revised analyses. Herd, sex, and birth type were included as prespecified fixed covariates in the machine-learning models and as fixed effects in the conventional mixed-model genome-wide association analysis. Summary statistics of the filtered phenotypic data are provided in Table 1.

### 2.2. Genomic DNA Extraction, Genotyping and Quality Control

Genomic DNA was extracted from whole blood samples using Qiacube HT auto-mated device using a commercial kit (Qiagen Blood kit, Hilden, Germany). The purified DNA was exposed to a quality/quantity check step to ensure that all samples passed the minimum genotyping requirements of A260/280 > 1.8; A260/230 > 1.5 and a sample amount of >20 ng/µL with a MultiSkan SkyHigh UV/VIS microplate spectrophotometer (ThermoFisher Scientific, Waltham, MA, USA). After checking for DNA integrity on gel electrophoresis, the samples proceeded to genotyping, which was implemented using the Illumina OvineSNP50 BeadChip (Illumina, San Diego, CA, USA) according to the manufacturer’s protocol.

The raw genotype dataset contained 64,734 SNP markers from 288 genotyped animals. Genotypes were encoded as 0 (AA), 1 (AB), and 2 (BB), while no-call genotypes were treated as missing values. Quality control was conducted sequentially. First, SNPs with a call rate below 90% were excluded, leaving 50,064 markers. Animals with more than 10% missing genotypes were then removed, resulting in 275 animals for genomic analysis. A minor allele frequency (MAF) threshold of 0.05 was subsequently applied, yielding 46,902 SNPs in the final genome-wide dataset.

For the machine-learning analyses, SNP-level missingness and MAF filtering were additionally performed within each outer training fold to ensure that information from the corresponding test fold did not influence preprocessing. Missing genotype values were imputed using SNP means estimated exclusively from the outer training data, and the same training-derived means were subsequently applied to the corresponding test set [18]. Thus, genotype imputation and fold-specific filtering were performed without using information from the outer test data.

### 2.3. Population Structure and Genomic Relatedness

Genomic relatedness among individuals was additionally characterized using a genomic relationship matrix (GRM). The GRM was subsequently incorporated into the conventional mixed-model GWAS to account for relatedness among animals.

A two-stage genome-wide association framework was employed, integrating regularized regression and non-linear machine learning algorithms to improve feature selection and trait prediction.

### 2.4. Machine-Learning-Based Genomic Prioritization and Prediction

**Figure 1:**
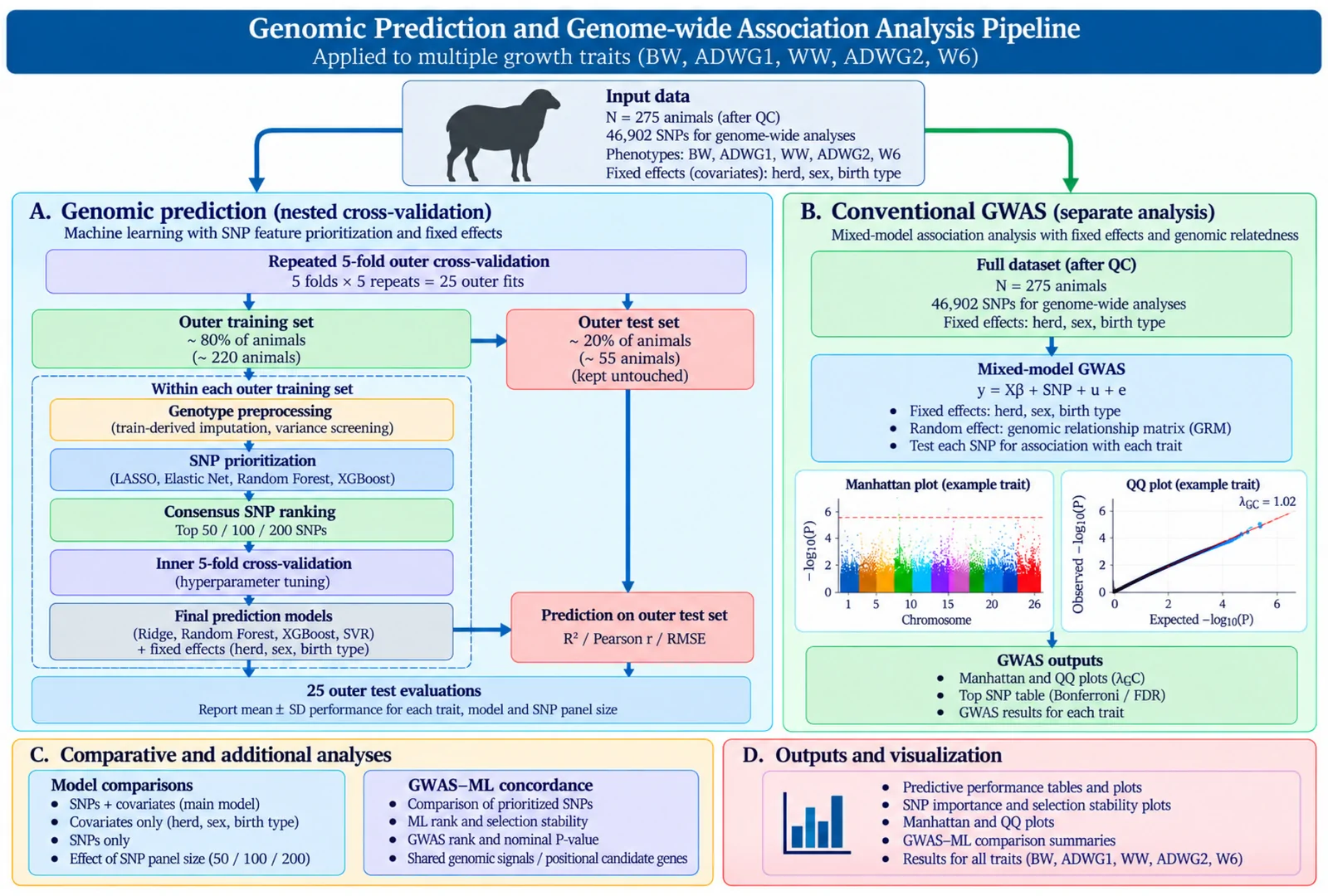
Overview of the analytical framework used for genomic prediction and genome-wide association analysis of growth traits in Central Anatolian Merino sheep.

Machine-learning analyses were designed to distinguish genomic feature prioritization and phenotype prediction from conventional SNP-wise association testing. The analytical framework combined repeated nested cross-validation, training-specific genomic preprocessing, multialgorithm SNP prioritization, alternative SNP-panel sizes, and predictive model evaluation (Figure 1). Herd, sex, and birth type were included as fixed covariates in both the SNP-prioritization and full predictive models.

Population structure and genomic relatedness were evaluated using the quality-controlled genotype data. Principal component analysis (PCA) was used to characterize major axes of genomic variation among animals, and the first principal components were examined in relation to herd, sex, and birth type to assess whether major genomic structure corresponded to these recorded population characteristics.

#### 2.4.1. Repeated Nested Cross-Validation Framework

Predictive performance was evaluated using repeated nested cross-validation. The outer cross-validation consisted of five folds repeated five times, resulting in 25 independent outer train–test evaluations for each trait. Within each repetition, approximately 80% of the animals were used for model development and approximately 20% were retained as an outer test set.

All supervised model-development procedures were repeated independently within each outer training set. These procedures included fold-specific SNP quality control, genotype imputation, variance-based dimensionality reduction, SNP prioritization, marker-panel construction, hyperparameter optimization, and final model fitting. The corresponding outer test phenotypes were not used during any of these procedures.

Hyperparameter optimization was performed using five-fold inner cross-validation restricted to the respective outer training set. No SNP panel was defined using the complete dataset before cross-validation; SNP prioritization and panel construction were repeated de novo within each outer training set. Consequently, each outer test set remained independent of the supervised feature-selection and model-tuning procedures used to generate its predictions.

Because the outer five-fold partitioning was repeated five times, an individual could occur in a test fold in one repetition and in a training fold in another repetition. However, each repetition and outer model fit was conducted independently, with no transfer of selected SNPs, preprocessing parameters, fitted model parameters, or hyperparameters between outer fits.

#### 2.4.2. SNP Prioritization and Marker-Panel Construction

Within each outer training set, SNPs passing fold-specific quality control were first ranked according to their genotype variance, and the 30,000 markers with the highest variance were retained as an initial dimensionality-reduction step. Genomic feature prioritization was subsequently performed using four complementary algorithms: LASSO regression) [19], Elastic Net regression [20], Random Forest [21], and XGBoost [22].

For LASSO (α = 1) and Elastic Net (α = 0.5), models were implemented using the *glmnet* package. The regularization parameter *λ* was optimized using five-fold cross-validation within the outer training data, and SNPs were ranked according to the absolute magnitude of their regression coefficients. Prespecified fixed covariates (herd, sex, and birth type) were included in the models but were excluded from the subsequent SNP-ranking calculation.

Random Forest-based prioritization was performed using 500 trees, permutation-based variable importance, a minimum node size of five, and an mtry value corresponding approximately to the square root of the number of predictors. Herd, sex, and birth type were retained as model covariates during feature prioritization. SNPs were ranked according to permutation importance.

XGBoost-based prioritization was performed using a squared-error objective, a learning rate of 0.05, maximum tree depth of 3, subsampling rate of 0.80, column-sampling rate of 0.50, and 200 boosting rounds. SNPs were ranked according to their Gain importance.

Because the four algorithms generate importance measures on different scales, method-specific SNP scores were converted to percentile ranks. A consensus score was then calculated as the mean percentile rank across LASSO, Elastic Net, Random Forest, and XGBoost. SNPs were ordered according to this consensus score, and the top 50, 100, and 200 markers were evaluated as alternative predictive panels. Evaluating three marker-panel sizes avoided reliance on a single arbitrarily selected number of SNPs and allowed assessment of the sensitivity of predictive performance to panel size.

#### 2.4.3. Predictive Models and Hyperparameter Optimization

For each of the 50-, 100-, and 200-SNP panels, four predictive algorithms were evaluated: Ridge regression, Random Forest (RF), Extreme Gradient Boosting (XGBoost), and Support Vector Regression (SVR) [21,22,23]. The full predictive models contained the selected SNP panel together with herd, sex, and birth type as fixed covariates.

Ridge regression was implemented using *glmnet* (Version: 5.1) with α = 0, and the optimal λ value was selected using five-fold inner cross-validation. Ridge regression was used as a predictive model rather than as a sparse feature-selection method.

Random Forest models were fitted using 500 trees. The number of candidate variables considered at each split (*mtry*) was tuned among values approximately corresponding to √p, p/3, and p/2, where p represents the number of predictors, while minimum node sizes of 3, 5, and 10 were evaluated. The splitting rule was variance reduction. Optimal settings were selected according to inner-cross-validation RMSE.

For XGBoost, the hyperparameter grid included 100 or 250 boosting rounds, maximum tree depths of 2 or 4, learning rates of 0.03 or 0.10, and column-sampling proportions of 0.70 or 1.00. The subsampling rate was fixed at 0.80, gamma at 0, and minimum child weight at 1. The parameter combination producing the lowest mean inner-cross-validation RMSE was selected.

SVR was implemented using a radial basis function kernel. Predictor variables were centered and scaled within the model-fitting procedure. The tuning grid included σ values of 0.001, 0.005, 0.01, and 0.05 and cost (C) values of 0.25, 1, 4, and 16. The optimal parameter combination was selected using five-fold inner cross-validation based on RMSE. The complete hyperparameter search grids and model-tuning settings are provided in Table S1, while the optimal hyperparameter combinations selected across outer training folds and SNP panels are reported in Table S2.

#### 2.4.4. Predictive Performance and SNP-Prioritization Stability

Predictions generated for each untouched outer test fold were evaluated using the coefficient of determination (R^2^), root mean square error (RMSE), and Pearson correlation coefficient (r) between observed and predicted phenotypes. Performance was calculated separately for each of the 25 outer evaluations, and the mean and standard deviation across outer folds were reported for each model and marker-panel size.

To provide reference models for interpreting predictive performance, a covariate-only model containing herd, sex, and birth type and SNP-only models containing the same ML-prioritized SNP panels without the fixed covariates were also evaluated. These analyses were treated as complementary model controls rather than as independent genomic association tests.

The stability of genomic prioritization was assessed across the 25 outer model-development sets. For each SNP, selection frequency was calculated as the proportion of outer fits in which the marker occurred among the consensus top-200 SNPs. This measure was used as a descriptive indicator of reproducibility of ML-based SNP prioritization and was not interpreted as a statistical significance test. SNPs with a selection stability ≥0.50 are reported in Table S3.

### 2.5. Conventional Mixed-Model Genome-Wide Association Analysis

In parallel with the machine-learning analyses, conventional genome-wide association analysis was performed separately for each growth trait to provide SNP-wise statistical association evidence. This analysis was deliberately separated from ML-derived feature prioritization because predictive importance and statistical association represent distinct analytical quantities.

For each trait, SNPs with >10% missing genotypes or MAF < 0.05 were excluded, and the remaining missing genotypes were replaced by marker-specific mean genotype values. A genomic relationship matrix was constructed from the quality-controlled genotype matrix using the *A.mat* function implemented in the *rrBLUP* R package (Version: 4.6.3).

Mixed-model GWAS was then performed using *rrBLUP*, with herd, sex, and birth type fitted as fixed effects and the genomic relationship matrix incorporated to account for relatedness among individuals. No additional principal components were included in the association model because genomic relatedness was modeled directly through the GRM. The P3D approximation was used for computational efficiency.

SNP-wise association probabilities were adjusted for multiple testing using both the Bonferroni procedure (α = 0.05/number of tested SNPs) and the Benjamini–Hochberg false discovery rate (FDR; q < 0.05). Manhattan and quantile–quantile (QQ) plots were generated for each trait. Genomic inflation factors (*λ*) were calculated from the median observed chi-square statistic relative to its expected value under the null hypothesis to evaluate potential inflation of the GWAS test statistics.

### 2.6. Exploratory Concordance Between ML Prioritization and GWAS

To facilitate biological interpretation, an additional full-data ML ranking was generated for each trait using the same four-algorithm consensus prioritization procedure described above. This analysis was used exclusively for exploratory SNP annotation and comparison with conventional GWAS results and was not used to estimate predictive performance.

Concordance between highly ranked ML markers and SNP-wise GWAS evidence was examined descriptively. Because the ML ranking and GWAS were derived from the same animals and phenotypes, overlap between the two approaches was not considered independent validation or evidence of genome-wide statistical significance. Accordingly, ML-derived feature importance, GWAS association probability, and cross-validation-based predictive performance were interpreted as distinct sources of evidence throughout the study.

### 2.7. Candidate Gene Annotation and Functional Enrichment

For exploratory positional annotation, genes located within ±100 kb of prioritized SNPs were identified using the Ensembl *Ovis aries* genome (Oar_v4.0). The ±100 kb interval was used as a standardized operational window for positional candidate-gene annotation and was not intended to represent an empirically estimated LD interval or a causal genomic region. Candidate gene identification was conducted using the biomaRt R package [24] referencing the Ensembl *Ovis aries* genome (Oar_v4.0). Genes identified within these regions were considered positional candidate genes rather than evidence of causal gene–trait relationships.

Genes overlapping or flanking the selected SNPs were extracted, and Ensembl Gene IDs were subjected to functional enrichment analysis using g:Profiler [25]. Enrichment was assessed across Gene Ontology Biological Process, Molecular Function, Cellular Component, KEGG, and Reactome pathways. Statistical significance was determined using Benjamini–Hochberg FDR correction (q < 0.05), and top terms were visualized using dot plots.

### 2.8. Software and Computational Resources

All genomic and statistical analyses were performed in R [26]. Data handling and visualization were conducted using data.table, readxl, dplyr, tidyr, ggplot2, openxlsx, and patchwork. Regularized regression models were implemented using glmnet; Random Forest analyses using ranger; XGBoost analyses using xgboost; SVR and model tuning using caret, kernlab, and e1071; and genomic relationship matrix construction and mixed-model GWAS using rrBLUP. Candidate-gene annotation and functional analyses were conducted using biomaRt and gprofiler2.

## 3. Results

### 3.1. Phenotypic and Genomic Data

Phenotypic records were available for 278 Central Anatolian Merino lambs for all five growth traits evaluated in the study. The dataset comprised 81 males and 197 females. Birth type was equally distributed between the two recorded categories (*n* = 139 each). The phenotypic traits included birth weight (BW), pre-weaning average daily gain (ADWG1), weaning weight (WW), post-weaning average daily gain (ADWG2), and six-month body weight (W6). Descriptive statistics for these traits are presented in Table 1.

Genotyping was initially available for 288 animals and 64,734 SNP markers. Following genotype quality control, 275 animals remained in the genomic dataset. SNP call-rate filtering retained 50,064 markers, and subsequent minor allele frequency filtering resulted in 46,902 SNPs for genome-wide analyses. These quality-controlled genomic data formed the basis for the population-structure analysis, machine-learning-based genomic prioritization, predictive modeling, and conventional mixed-model GWAS.

### 3.2. Population Structure and Genomic Relatedness

**Figure 2:**
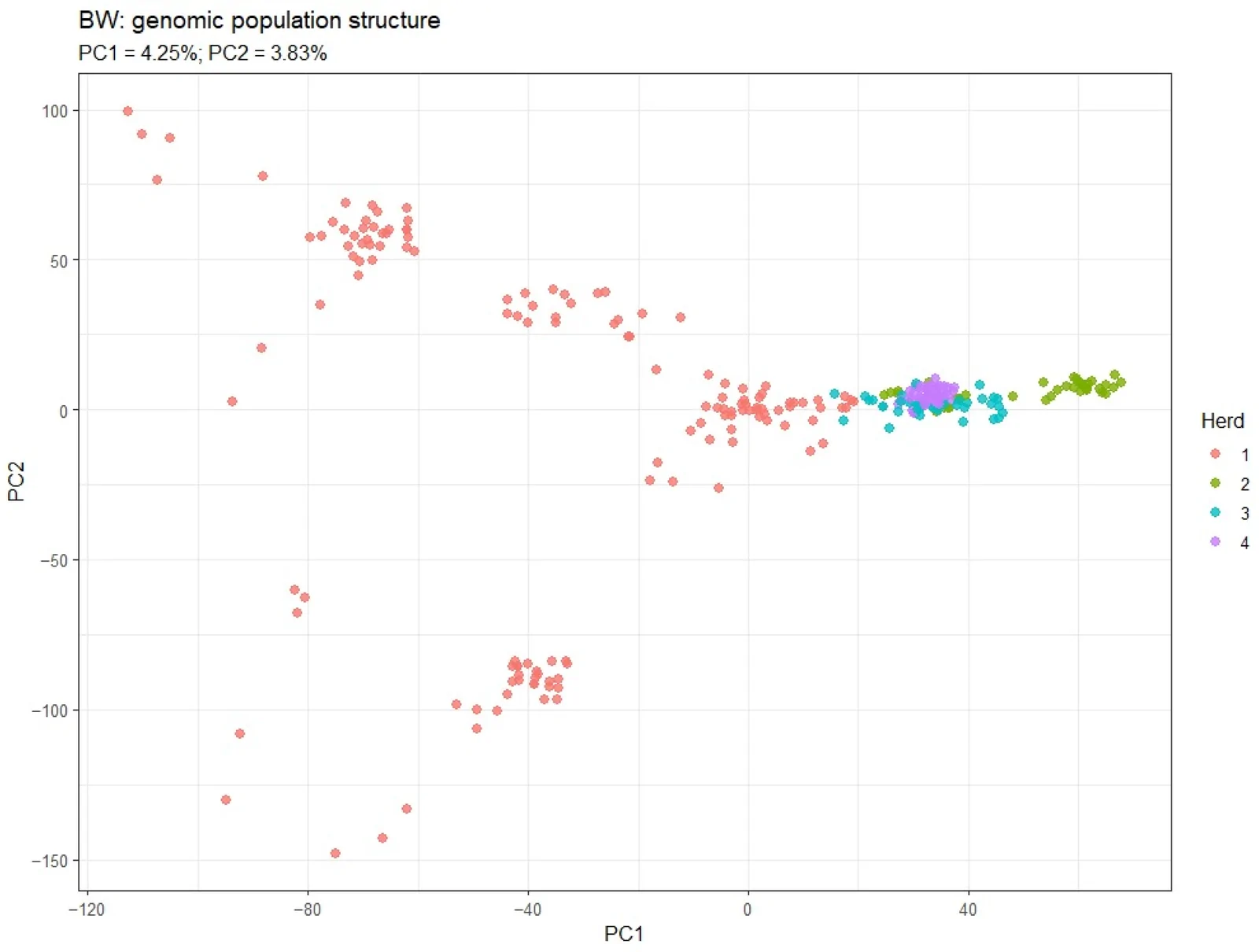
Principal component analysis of the quality-controlled genomic data from Central Anatolian Merino sheep.

Principal component analysis of the quality-controlled genotype data revealed detectable genomic structure within the study population (Figure 2). The first and second principal components explained 4.36% and 4.02% of the total genomic variation, respectively. Visualization of the first two principal components according to herd membership indicated herd-associated clustering, although substantial overlap among animals was also observed. In contrast, no comparable separation was evident according to sex or birth type.

Genomic relatedness also varied among individuals, consistent with the breeding structure of the study population. Therefore, herd was included as a fixed effect in subsequent analyses, while the genomic relationship matrix was incorporated into the conventional mixed-model GWAS to account for relatedness among animals.

### 3.3. Predictive Performance Under Repeated Nested Cross-Validation

**Figure 3:**
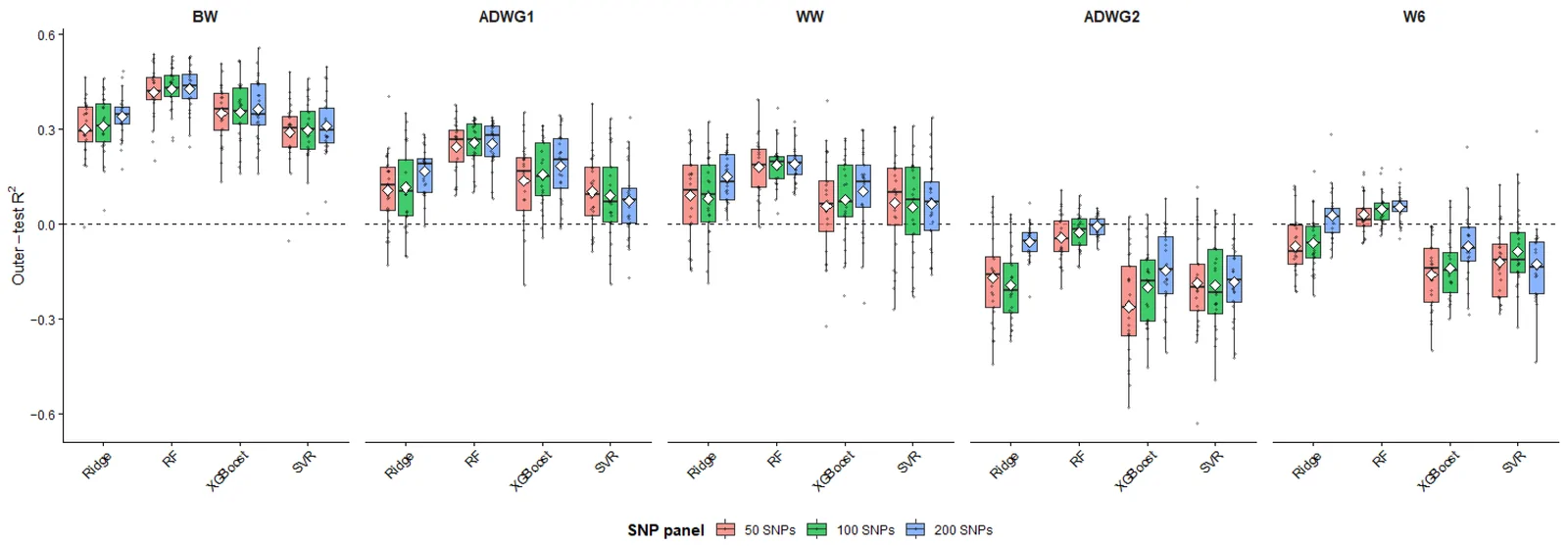
Predictive performance of Ridge regression, Random Forest, XGBoost, and Support Vector Regression using 50-, 100-, and 200-SNP panels across five growth traits.

Predictive performance was evaluated across 25 outer test folds generated by five-fold cross-validation repeated five times. Across the combinations of predictive algorithms and SNP-panel sizes, performance differed substantially among growth traits (Table 2; Figure 3). The strongest predictive performance was observed for BW, followed by ADWG1 and WW, whereas predictive performance was limited for W6 and particularly ADWG2.

For BW, the highest mean predictive performance was obtained using Random Forest with the 100-SNP panel (R^2^ = 0.426 ± 0.068; r = 0.661 ± 0.053; RMSE = 0.800 ± 0.062 kg). Random Forest with 100 SNPs also provided the highest performance for ADWG1 (R^2^ = 0.256 ± 0.070; r = 0.525 ± 0.073; RMSE = 0.0616 ± 0.0032 kg/day). For WW, the best-performing combination was Random Forest with 200 SNPs (R^2^ = 0.188 ± 0.058; r = 0.466 ± 0.089; RMSE = 5.797 ± 0.313 kg).

Predictive performance was substantially lower for the two later-growth traits. Random Forest with the 200-SNP panel yielded a mean R^2^ of −0.008 ± 0.033 for ADWG2, with r = 0.114 ± 0.069 and RMSE = 0.0403 ± 0.0052 kg/day. For W6, the corresponding Random Forest model achieved R^2^ = 0.053 ± 0.049, r = 0.238 ± 0.103, and RMSE = 6.834 ± 0.630 kg.

Thus, under the repeated nested validation framework, genomic predictive performance showed a marked trait-dependent pattern, with substantially greater predictive ability for BW and early growth than for post-weaning growth traits. The substantially lower performance estimates obtained under nested validation compared with those reported in the original analysis also demonstrate the importance of performing SNP prioritization and model optimization exclusively within the training data.

### 3.4. Stability of ML-Based SNP Prioritization

The reproducibility of SNP prioritization was evaluated across the 25 outer training sets by calculating the proportion of outer fits in which each marker was included in the top-200 consensus SNP panel. Selection stability differed among traits, with a larger number of recurrently prioritized markers for BW than for the other growth traits.

For BW, 26 SNPs were selected in at least 50% of the outer training sets, including seven markers with a selection stability of at least 0.75. The highest stability observed for BW was 0.88. For ADWG1 and WW, 14 and 13 SNPs, respectively, reached a stability of at least 0.50, with two markers for each trait exceeding 0.75. Maximum selection stability was 0.84 for ADWG1 and 0.92 for WW.

Fewer recurrently prioritized markers were observed for the later-growth traits. Four SNPs reached a stability of at least 0.50 for ADWG2, with a maximum stability of 0.64, whereas eight SNPs reached this threshold for W6, including two with stability ≥ 0.75 and a maximum stability of 0.92.

**Figure 4:**
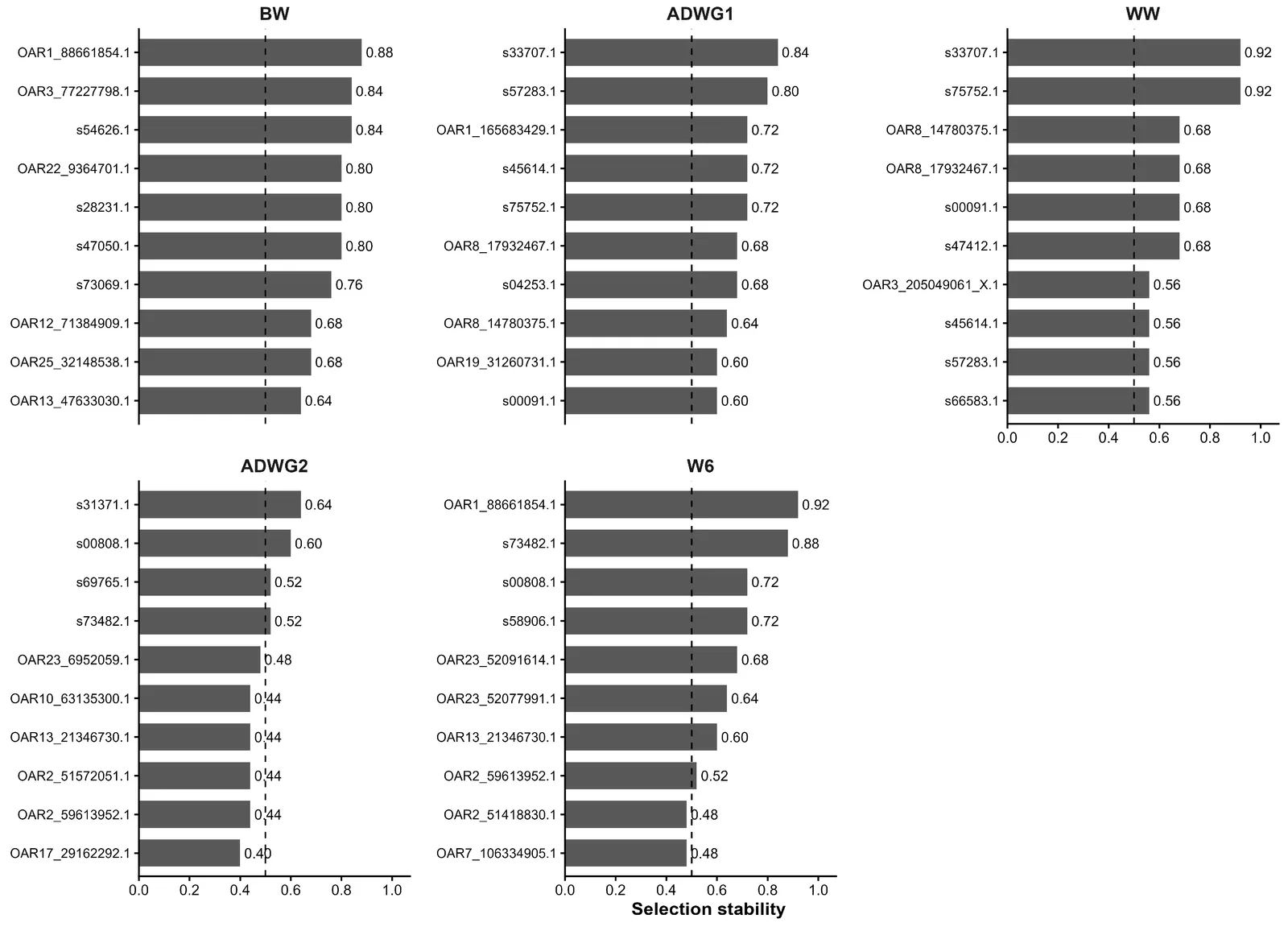
Stability of machine-learning-based SNP prioritization across repeated outer training sets. The ten SNPs with the highest selection stability are shown for each growth trait. Selection stability represents the proportion of the 25 outer training sets in which an SNP was included in the top-200 consensus panel. The dashed vertical line at 0.50 is provided as a descriptive reference and does not represent a statistical significance threshold. BW, birth weight; ADWG1, pre-weaning average daily gain; WW, weaning weight; ADWG2, post-weaning average daily gain; W6, six-month body weight.

The highest-ranking recurrently prioritized SNPs for each trait are shown in Figure 4. Selection stability represents the reproducibility of ML-based prioritization across resampled training datasets and should not be interpreted as a test of statistical significance.

### 3.5. Conventional Mixed-Model Genome-Wide Association Analysis

**Figure 5:**
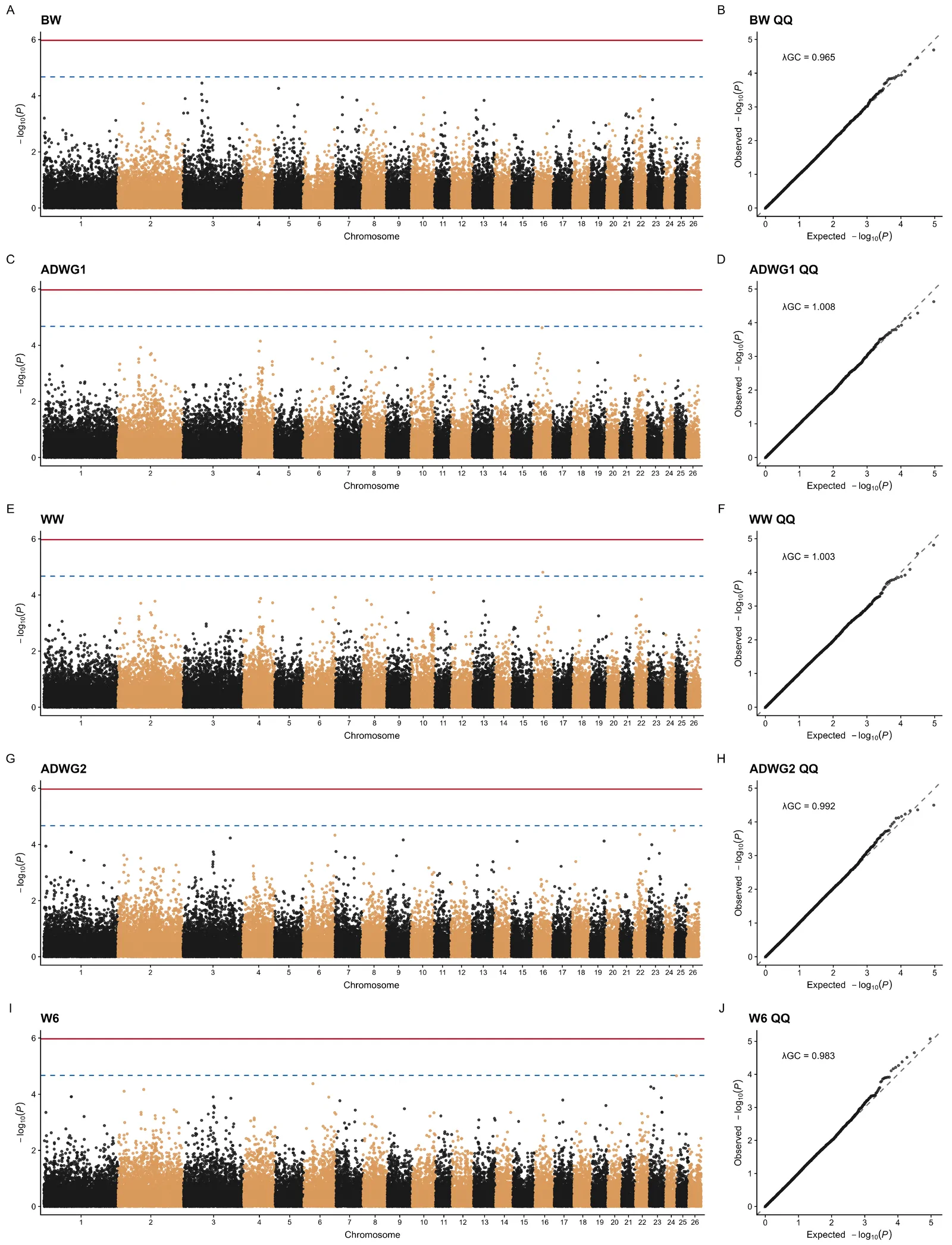
Manhattan and quantile–quantile (QQ) plots from conventional mixed-model genome-wide association analyses of five growth traits in Central Anatolian Merino sheep. Panels (**A**,**B**) represent birth weight (BW), (**C**,**D**) pre-weaning average daily gain (ADWG1), (**E**,**F**) weaning weight (WW), (**G**,**H**) post-weaning average daily gain (ADWG2), and (**I**,**J**) six-month body weight (W6). Panels (**A**,**C**,**E**,**G**,**I**) show Manhattan plots, whereas panels (**B**,**D**,**F**,**H**,**J**) show the corresponding QQ plots. In the Manhattan plots, alternating black and orange points distinguish SNPs located on consecutive chromosomes. The solid red horizontal line indicates the Bonferroni-corrected genome-wide significance threshold (*p* < 1.07 × 10^−6^), whereas the dashed blue line indicates the genome-wide suggestive threshold (*p* < 2.13 × 10^−5^).

Conventional mixed-model GWAS was performed for the five growth traits using 46,902 quality-controlled SNPs. No SNP reached genome-wide significance after Bonferroni or FDR correction; however, several suggestive and chromosome-wide signals were identified (Table 3; Figure 5).

Three markers exceeded the genome-wide suggestive threshold (*p* < 2.13 × 10^−5^): s75752.1 on chromosome 22 for BW (*p* = 2.05 × 10^−5^), s10813.1 on chromosome 16 for WW (*p* = 1.55 × 10^−5^), and the unplaced marker OARUn.501_205348.1 for W6 (*p* = 8.46 × 10^−6^). Notably, s10813.1 also reached chromosome-wide significance for ADWG1 (*p* = 2.38 × 10^−5^). Similarly, s10591.1 on chromosome 24 showed chromosome-wide signals for both ADWG2 (*p* = 3.18 × 10^−5^) and W6 (*p* = 2.21 × 10^−5^). An additional chromosome-wide signal, OAR22_26079325.1, was identified for ADWG2 (*p* = 4.38 × 10^−5^).

Genomic inflation factors were close to unity across traits (λGC = 0.965–1.008), indicating no marked inflation of the GWAS test statistics. The recurrence of s10813.1 across ADWG1 and WW and s10591.1 across ADWG2 and W6 was interpreted as shared genomic signals rather than evidence of pleiotropy.

### 3.6. Concordance Between ML Prioritization and GWAS Evidence

Several genomic signals shared between related growth traits showed concordance between ML-based SNP prioritization and conventional GWAS (Table 4). For ADWG1 and WW, s04253.1 showed consistent evidence across both analytical approaches, ranking 19th and 36th in ML prioritization and 10th and 20th in GWAS, respectively. s64483.1 showed a similar pattern, ranking 20th and 16th in ML prioritization and 27th and 31st in GWAS for ADWG1 and WW, respectively. s57283.1, s69091.1, and OAR1_165683429.1 were also recurrently prioritized by ML, although their relative ML and GWAS rankings differed between the two traits.

Concordance was particularly apparent for ADWG2 and W6. s00808.1 ranked first in ML prioritization and eighth in GWAS for ADWG2, with a selection stability of 0.60, and ranked fifth and sixth, respectively, for W6, with a stability of 0.72. Similarly, s73482.1 ranked fourth in ML prioritization and 15th in GWAS for ADWG2 and 11th and 13th, respectively, for W6; its selection stability reached 0.88 for W6. s69765.1 also showed concordant evidence, particularly for ADWG2, where it ranked 14th in ML prioritization and sixth in GWAS. OAR23_52077991.1 was highly ranked by ML for both ADWG2 and W6, although stronger GWAS support was observed for W6.

Overall, these findings indicate convergence between ML-based genomic prioritization and nominal GWAS evidence for a subset of markers across related growth traits. However, because both analytical approaches were applied to the same study population and none of these SNPs reached genome-wide significance after multiple-testing correction, these concordant signals were considered exploratory rather than independently validated associations.

### 3.7. Positional Candidate Genes

Positional annotation of the shared genomic signals identified several genes located within or near the prioritized SNPs (Table 5). Among the signals shared between ADWG1 and WW, candidate regions included RASD2 and MB near s57283.1, PRMT1 at s69091.1, CERKL and NEUROD1 near s04253.1, TNC near s64483.1, and CHMP2B and POU1F1 near OAR1_165683429.1.

For signals shared between ADWG2 and W6, positional candidates included ERCC6L2, SSH2, CDCP1, CLEC3B, EXOSC7, ZDHHC3, RNF165, LOXHD1, ST8SIA5, PIAS2, DYM, and LINGO2. Several of these SNPs were located within annotated genes, whereas others were located in nearby genomic regions.

These genes were considered positional candidates associated with the prioritized genomic regions rather than functionally or causally validated genes. Their potential biological relevance to growth-related processes is considered further in the Discussion.

## 4. Discussion

The growth traits of lambs play a critical role in the sheep industry, as they determine the economic value of the animals and serve as a key factor in ensuring the quality of lamb production. This study integrated machine-learning-based genomic prioritization and prediction with conventional mixed-model genome-wide association analysis to identify genomic signals and positional candidate genes associated with key growth traits in Central Anatolian Merino sheep. The analytical framework incorporated repeated nested cross-validation, training-specific SNP prioritization, and conventional GWAS as a complementary source of SNP-wise association evidence. The results underscore the relevance of using machine learning (ML) to investigate and prioritize genomic signals underlying polygenic traits with complex genetic architectures and environmental interactions. Conventional GWAS methods, though interpretable, often fall short in identifying polygenic signals when SNP effects are non-additive or when epistasis is prevalent [27]. In the present study, predictive performance differed substantially among traits, with the strongest performance observed for BW and ADWG1, followed by WW, whereas predictive ability was considerably weaker for ADWG2 and W6. This pattern indicates that the genomic information captured by the evaluated ML models differed across developmental stages rather than demonstrating uniformly high predictive performance across traits. 

Most growth phenotypes are quantitative traits and are regulated by multiple genes, and, research on the relationship between genetics and growth traits began decades ago. As early as the 2000s, researchers identified a QTL significantly associated with BW linked to *BMPR1B* [28] and a QTL for ADG linked to *MSTN* [29]. Subsequently, a growing number of studies detected thousands of QTLs across the sheep genome associated with numerous economically important traits (Sheep QTLdb); however, compared to other livestock species, the number of reported QTLs in sheep remains relatively limited [30]. Even though there has been substantial research in this field, the available evidence is still not enough to give a complete explanation of the genetic mechanisms underlying these traits, and additional populations and genomic studies remain valuable for further characterizing their complex genetic architecture.

Weaning weight is a critical performance metric in sheep production, acting as an early predictor of lamb development potential, maternal capability, and overall flock output. Increased weaning weights correlate with increased survival rates, heightened tolerance to environmental stressors, and superior post-weaning growth performance. Moreover, weaning weight indicates management effectiveness, nutritional status, and ewe body condition, and is extensively utilized as a selection criterion in genetic enhancement initiatives [31]. Thus, enhancing weaning weight immediately enhances biological efficiency and economic sustainability in lamb production systems. Cross-trait comparison of the revised genomic results showed recurrent genomic signals between adjacent developmental stages, particularly between ADWG1–WW and ADWG2–W6 (Table 4). These recurrent signals may reflect genomic regions contributing to correlated or temporally related growth processes; however, their occurrence across traits should not be interpreted as evidence of pleiotropy without formal multivariate or genetic-correlation analyses. Several recurrently prioritized SNPs showed concordant ML prioritization and nominal GWAS evidence for ADWG1 and WW.

The SNP s57283.1 (OAR3: rs430517267; 178.6 Mb) located ~63 kb from *RASD2* and *MB* (Table 5), suggesting potential relevance to growth-related signaling and metabolic regulation. Myoglobin is a cytoplasmic hemoprotein that is restricted to cardiomyocytes and oxidative skeletal myofibers and facilitates oxygen delivery during periods of high metabolic demand [32]. There are not many studies in livestock species investigating associations between MB genotypes and growth traits. Among them, genetic correlation between myoglobin levels and growth traits was detected in Merino sheep [33]. The SNP s69091.1 (OAR14: rs426119338; 54.9 Mb) was located within *PRMT1*, a gene implicated in epigenetic regulation and muscle cell differentiation, indicating a potential role in transcriptional control of growth. Protein arginine methyltransferases (PRMTs) have emerged as important regulators of skeletal muscle metabolism and regeneration [34]. *PRMT1*-deficient muscle stem cells were found to be unable to terminate the myogenic differentiation program, leading to skeletal muscle regeneration failure [35]. Another notable finding was the recurrent prioritization of SNP s64483.1 (OAR2: rs410036333; 8.6 Mb) across ADWG1 and WW. The *TNC* gene is an extracellular matrix protein that is involving in skeletal muscle development from embryonic stage to senescence [36]. Similarly, in pigs, variations in *TNC* have been linked to carcass traits and meat quality [37], suggesting that this genomic region may warrant further investigation in relation to early growth in sheep. Furthermore, OAR1_165683429.1 SNP located on chromosome 1 (rs401183514, 153.8 Mb), near *CHMP2B* and *POU1F1*, was recurrently prioritized for ADWG1 and WW (Table 4 and Table 5). The *POU1F1* is a member of the POU-domain family and functions as an important transcriptional regulator of growth hormone expression; polymorphisms in this gene have previously been associated with growth traits in sheep [38] and goats [39].

Our study also found that SNPs located within or near genes including *ERCC6L2*, *SSH2*, *DYM*, and *LINGO2* were among the recurrent genomic signals observed across ADWG2 and W6 (Table 4 and Table 5). The *ERCC6L2* gene encodes a helicase-like protein belonging to the Snf2 family [40] which is involved in mechanical remodeling of chromatin structure and has regulatory functions relevant to mammalian development, including myoblast proliferation [41]. Another myoblast regulator represented among the positional candidates was slingshot protein phosphatase 2 (*SSH2*) which encodes a protein tyrosine phosphatase [42]. Furthermore, the identified *DYM* gene which encodes a Golgi-associated protein, plays essential role in intracellular trafficking. Variants in the *DYM* gene were reported to be associated with Dyggve–Melchior–Clausen syndrome in humans, which is characterized by skeletal defects such as short limbs and trunk and valgus deformity of the knees [43,44]. These findings suggest that *DYM* represents a biologically plausible positional candidate in a genomic region prioritized for growth traits, although the present results do not establish a causal role in skeletal development or growth. *LINGO2* encodes a transmembrane protein belonging to the LINGO/LERN protein family which contains four homologs in the human genome (LINGO1-4). Only a few studies have been performed specifically concerning the function of *LINGO2*. Human *LINGO2* variants were associated with neurodegenerative diseases such as Essential tremor and Parkinson disease [45,46]. No studies have directly established an association between *LINGO2* variants and growth traits in livestock; however, one study reported an association between *LINGO2* and body mass in an elderly human population [47]. Therefore, its potential relationship with growth performance in sheep remains speculative and requires independent validation.

## 5. Conclusions

This study demonstrates the utility of integrating machine-learning-based genomic prioritization and prediction with conventional mixed-model GWAS for investigating the genetic architecture of growth traits in Central Anatolian Merino sheep. Using repeated nested cross-validation, in which SNP prioritization, marker-panel construction, model tuning, and prediction were performed independently within the training data, the study provided a more rigorous assessment of predictive performance and SNP prioritization. Predictive performance varied across traits and was strongest for birth weight (BW) and pre-weaning average daily gain (ADWG1), moderate for weaning weight (WW), and substantially weaker for post-weaning average daily gain (ADWG2) and six-month body weight (W6). The analysis also identified recurrent genomic signals across related growth traits, particularly between ADWG1–WW and ADWG2–W6. These shared signals should be regarded as evidence of genomic concordance across related traits rather than as evidence of pleiotropy. Several biologically plausible positional candidate genes, including *PRMT1*, *TNC*, *POU1F1*, *RASD2*, *ERCC6L2*, *SSH2*, and *DYM*, were highlighted, with reported biological functions related to muscle development, skeletal growth, metabolism, and cellular regulation. However, these positional annotations do not establish causal relationships between the identified genes and the evaluated growth traits.

Overall, these findings support the complementary use of machine-learning-based genomic prioritization and conventional GWAS for investigating complex, polygenic growth traits in livestock. Although no SNP reached genome-wide significance after multiple-testing correction, concordance between ML prioritization and nominal GWAS evidence was observed for a subset of genomic signals. Given the relatively small study population and the absence of an independent external validation population, the identified SNPs and positional candidate genes should be considered exploratory candidates requiring further validation. Independent studies in larger and genetically distinct sheep populations will be necessary before their potential application in genomic selection programs can be established.

## Figures and Tables

**Table 1:** Descriptive statistics for growth traits.

Trait	N	Mean	SE Mean	Min	Max	SD	CV (%)
BW (kg)	278	4.653	0.0635	2.44	6.86	1.059	22.76
ADWG1 (kg/day)	278	0.2501	0.00431	0.085	0.441	0.0719	28.71
WW (kg)	278	27.160	0.388	12.38	43.47	6.463	23.80
ADWG2 (kg/day)	278	0.1816	0.00243	0.0546	0.434	0.0405	22.28
W6 (kg)	278	37.344	0.452	14.28	82.30	7.539	20.19

**Table 2:** Predictive performance of the best-performing model and SNP-panel combination for each growth trait under five-fold outer cross-validation repeated five times (25 outer test evaluations).

Trait	Model	SNP Panel	R^2^, Mean ± SD	Pearson’s r, Mean ± SD	RMSE, Mean ± SD
BW (kg)	Random Forest	100	0.426 ± 0.068	0.661 ± 0.053	0.800 ± 0.062
ADWG1 (kg/day)	Random Forest	100	0.256 ± 0.070	0.525 ± 0.073	0.0616 ± 0.0032
WW (kg)	Random Forest	200	0.188 ± 0.058	0.466 ± 0.089	5.797 ± 0.313
ADWG2 (kg/day)	Random Forest	200	−0.008 ± 0.033	0.114 ± 0.069	0.0403 ± 0.0052
W6 (kg)	Random Forest	200	0.053 ± 0.049	0.238 ± 0.103	6.834 ± 0.630

**Table 3:** Suggestive genome-wide and chromosome-wide association signals identified for growth traits in Central Anatolian Merino sheep.

Trait	SNP	Chr	Position (bp)	*p*-Value	FDR	Evidence
BW	s75752.1	22	25,327,313	2.05 × 10^−5^	0.627	GW
ADWG1	s10813.1	16	34,212,515	2.38 × 10^−5^	0.724	CW
WW	s10813.1	16	34,212,515	1.55 × 10^−5^	0.651	GW
WW	OAR10_81003685.1	10	81,003,685	2.78 × 10^−5^	0.651	CW
ADWG2	s10591.1	24	43,579,432	3.18 × 10^−5^	0.518	GW
ADWG2	OAR22_26079325.1	22	26,079,325	4.38 × 10^−5^	0.518	CW
W6	s10591.1	24	43,579,432	2.21 × 10^−5^	0.456	GW

**Table 4:** Re-evaluation of shared genomic signals using ML-based SNP prioritization and conventional GWAS across related growth traits.

Shared Signal	Trait	SNP	Chr.	Position (bp)	rs ID	ML Rank	Selection Stability	GWAS Rank	GWAS *p*-Value
ADWG1–WW	ADWG1	s57283.1	3	178,572,430	rs430517267	11	0.80	70	1.55 × 10^−3^
ADWG1–WW	WW	s57283.1	3	178,572,430	rs430517267	474	0.56	186	3.93 × 10^−3^
ADWG1–WW	ADWG1	s69091.1	14	54,999,162	rs426119338	5	0.48	130	2.69 × 10^−3^
ADWG1–WW	WW	s69091.1	14	54,999,162	rs426119338	8	0.52	193	4.15 × 10^−3^
ADWG1–WW	ADWG1	s04253.1	2	126,954,461	rs413179794	19	0.68	10	1.96 × 10^−4^
ADWG1–WW	WW	s04253.1	2	126,954,461	rs413179794	36	0.48	20	5.10 × 10^−4^
ADWG1–WW	ADWG1	s64483.1	2	8,585,300	rs410036333	20	0.52	27	4.69 × 10^−4^
ADWG1–WW	WW	s64483.1	2	8,585,300	rs410036333	16	0.48	31	6.85 × 10^−4^
ADWG1–WW	ADWG1	OAR1_165683429.1	1	153,765,895	rs401183514	15	0.72	92	2.02 × 10^−3^
ADWG1–WW	WW	OAR1_165683429.1	1	153,765,895	rs401183514	478	0.44	245	5.22 × 10^−3^
ADWG2–W6	ADWG2	OAR2_31107932.1	2	30,073,969	rs161902751	12	0.20	117	2.36 × 10^−3^
ADWG2–W6	W6	OAR2_31107932.1	2	30,073,969	rs161902751	36	0.28	253	5.13 × 10^−3^
ADWG2–W6	ADWG2	OAR11_21353989.1	11	20,634,566	rs430250819	13	0.24	62	1.08 × 10^−3^
ADWG2–W6	W6	OAR11_21353989.1	11	20,634,566	rs430250819	9	0.40	38	5.93 × 10^−4^
ADWG2–W6	ADWG2	s69765.1	19	54,119,454	rs408790083	14	0.52	6	7.54 × 10^−5^
ADWG2–W6	W6	s69765.1	19	54,119,454	rs408790083	84	0.36	17	2.53 × 10^−4^
ADWG2–W6	ADWG2	s73482.1	23	46,581,238	rs161387042	4	0.52	15	2.07 × 10^−4^
ADWG2–W6	W6	s73482.1	23	46,581,238	rs161387042	11	0.88	13	1.34 × 10^−4^
ADWG2–W6	ADWG2	s00808.1	23	17,445,462	rs414971578	1	0.60	8	1.02 × 10^−4^
ADWG2–W6	W6	s00808.1	23	17,445,462	rs414971578	5	0.72	6	6.18 × 10^−5^
ADWG2–W6	ADWG2	OAR23_52077991.1	23	49,068,887	rs405253663	2	0.32	128	2.58 × 10^−3^
ADWG2–W6	W6	OAR23_52077991.1	23	49,068,887	rs405253663	4	0.64	24	4.40 × 10^−4^
ADWG2–W6	ADWG2	OAR2_103024142.1	2	95,689,756	rs414707093	27,147	0.08	53	9.23 × 10^−4^
ADWG2–W6	W6	OAR2_103024142.1	2	95,689,756	rs414707093	83	0.32	7	6.80 × 10^−5^

**Table 5:** Positional annotation of shared genomic signals identified across related growth traits.

Shared Traits	SNP	rs ID	Chr.	Position (bp)	Associated Gene(s)	Distance
ADWG1, WW	s57283.1	rs430517267	3	178,572,430	*RASD2*, *MB*	~63 kbp
ADWG1, WW	s69091.1	rs426119338	14	54,999,162	*PRMT1*	Within
ADWG1, WW	s04253.1	rs413179794	2	126,954,461	*CERKL*, *NEUROD1*	~47.3 kbp
ADWG1, WW	s64483.1	rs410036333	2	8,585,300	*ENSOARG00000005910*, *TNC*	~54.7 kbp
ADWG1, WW	OAR1_165683429.1	rs401183514	1	153,765,895	*CHMP2B*, *POU1F1*	~90 kbp
ADWG2, W6	OAR2_31107932.1	rs161902751	2	30,073,969	*ERCC6L2*	Within
ADWG2, W6	OAR11_21353989.1	rs430250819	11	20,634,566	*SSH2*	Within
ADWG2, W6	s69765.1	rs408790083	19	54,119,454	*CDCP1*, *CLEC3B*, *EXOSC7*, *ZDHHC3*	~7.8 kbp
ADWG2, W6	s73482.1	rs161387042	23	46,581,238	*RNF165*, *LOXHD1*, *ST8SIA5*, *PIAS2*	Within
ADWG2, W6	s00808.1	rs414971578	23	17,445,462	*LOC105604415*	~4.3 kbp
ADWG2, W6	OAR23_52077991.1	rs405253663	23	49,068,887	*DYM*	Within
ADWG2, W6	OAR2_103024142.1	rs414707093	2	95,689,756	*LINGO2*	Within

Note: Chr., chromosome. Genomic positions, rs identifiers, associated genes, and SNP-to-gene distances correspond to the marker annotation used for positional candidate-gene analysis. “Within” indicates that the SNP was located within the annotated gene, whereas distances are reported for genes located near the corresponding SNP.

## Data Availability

The datasets used during the current study are available from the corresponding author on reasonable request.

## Supplementary Materials

The following supporting information can be downloaded at https://www.mdpi.com/article/10.3390/ani16203243/s1, Table S1: Hyperparameter search grids and model-tuning settings; Table S2: Optimal hyperparameter settings selected across outer cross-validation folds and SNP-panel sizes; Table S3: Recurrently prioritized SNPs with selection stability ≥0.50 across the 25 outer model-development sets.
